# Regulation of influenza virus replication by Wnt/β-catenin signaling

**DOI:** 10.1371/journal.pone.0191010

**Published:** 2018-01-11

**Authors:** Sunil More, Xiaoyun Yang, Zhengyu Zhu, Gayan Bamunuarachchi, Yujie Guo, Chaoqun Huang, Keith Bailey, Jordan P. Metcalf, Lin Liu

**Affiliations:** 1 The Lundberg-Kienlen Lung Biology and Toxicology Laboratory, Department of Physiological Sciences, Oklahoma State University, Stillwater, Oklahoma, United States of America; 2 Oklahoma Center for Respiratory and Infectious Diseases, Stillwater, Oklahoma, United States of America; 3 Department of Veterinary Pathobiology, Oklahoma State University, Stillwater, Oklahoma, United States of America; 4 Oklahoma Animal Disease Diagnostic Laboratory, Stillwater, Oklahoma, United States of America; 5 Pulmonary and Critical Care Division, Department of Medicine, University of Oklahoma Health Sciences Center, Oklahoma City, Oklahoma, United States of America; Mayo Clinic Minnesota, UNITED STATES

## Abstract

Wnt/β-catenin signaling is an essential pathway in cell cycle control. Dysregulation of the Wnt/β-catenin signaling pathway during viral infection has been reported. In this study, we examined the effect of modulating Wnt/β-catenin signaling during influenza virus infection. The activation of the Wnt/β-catenin pathway by Wnt3a increased influenza virus mRNA and virus production in *in vitro* in mouse lung epithelial E10 cells and mRNA expresson of influenza virus genes *in vivo* in the lungs of mice infected with influenza virus A/Puerto Rico/8/34. However, the inhibition of Wnt/β-catenin signaling by iCRT14 reduced virus titer and viral gene expression in human lung epithelial A549 cells and viral replication in primary mouse alveolar epithelial cells infected with different influenza virus strains. Knockdown of β-catenin also reduced viral protein expression and virus production. iCRT14 acts at the early stage of virus replication. Treatment with iCRT14 inhibited the expression of the viral genes (vRNA, cRNA and mRNA) evaluated in this study. The intraperitoneal administration of iCRT14 reduced viral load, improved clinical signs, and partially protected mice from influenza virus infection.

## Introduction

Influenza virus is a single-stranded, negative-sense RNA virus belonging to the *Orthomyxoviridae* family. It has 8 genome segments that encode 10–12 different proteins [1]. Based on the antigenic differences in the nucleoprotein (NP) and M1 matrix proteins (MP), influenza viruses are classified as types A, B and C. For type A viruses, further subtyping is based on the antigenicity of hemagglutinin (HA) and neuraminidase (NA). Sixteen HA (H1—H16) and 9 NA (N1—N9) subtypes have been recognized. The influenza virion contains negative-sense RNA (vRNA). The synthesis of influenza virus RNA occurs in three steps: (i) vRNA is transcribed into mRNA by a cap-snatching mechanism via RNA-dependent RNA polymerase; (ii) intermediate cRNA is synthesized from vRNA; and (iii) cRNA is copied to full-length negative-sense vRNA for encapsulation in new progeny virus particles [2,3].

The replication mechanism of the virus is error-prone, resulting in mutations in the viral genome [4,5]. Whereas antigenic drift results from point mutations in surface antigens during viral replication, antigenic shift occurs when genetic reassortment between two different influenza viruses infecting the same cell takes place. Antigenic shift causes the emergence of pandemic strains of virus that can potentially infect very large populations of humans and animals [6].

The use of vaccines during viral pandemics has many limitations, including the time required for vaccine production, the efficacy of the vaccine in the target population and access to the vaccine during pandemics [7]. Thus, patient care depends upon the availability of effective antiviral drugs. However, emerging influenza viruses often become resistant to the available antiviral drugs because these drugs target the viral proteins [8,9]. Due to its small genome size, influenza virus is dependent on the host cell machinery for its replication and packaging. Many host factors utilized by influenza virus at various stages of the viral life cycle have been identified [10,11]. The development of antiviral drugs that target these factors can overcome the development of drug resistance that limits the use of current anti-influenza virus drugs.

The Wnt/β-catenin signaling pathway plays an important role in cellular development and differentiation and has been implicated in developmental diseases and cancer [12]. This pathway is activated by the binding of Wnt ligands to the Frizzled receptor. The central component of the pathway, β-catenin has two functions, one as an adaptor linking cadherins to the cytoskeleton at the cell membrane and another as a transcription factor. In the absence of Wnt ligands such as Wnt3a, cytoplasmic β-catenin forms a complex with adenomatous polyposis coli (APC), axin and glycogen synthase kinase 3β (GSK3β). Once bound, β-catenin is phosphorylated, then ubiquitinylated by the β-transducin repeat-containing protein and degradated via the proteasome. When Wnt ligands are present, the complex is destabilized, leading to the accumulation and nuclear translocation of cytosolic β-catenin. In the nucleus, β-catenin interacts with the T cell factor/lymphoid enhancer binding factor 1 (TCF/LEF) transcription factor to activate Wnt target genes along with other co-factors, CBP/p300. iCRT14 is an inhibitor of Wnt/β-catenin signaling that disrupts the direct interaction between β-catenin and TCF4/LEF1[13]. This inhibitor also prevents the binding of TCF4 to DNA; however, iCRT14 does not affect non-canonical Wnt signaling or other pathways such as Hedgehog, JAK/STAT or notch signaling [13].

Several studies have documented the interaction of the Wnt/β-catenin signaling pathway with viruses, including the human immunodeficiency virus (HIV) and the hepatitis C virus (HCV). HIV’s negative regulatory factor (Nef) interacts with β-catenin to inhibit Wnt/β-catenin signaling [14]. The core protein and nonstructural NS5A protein of HCV activate the Wnt/β-catenin signaling pathway [15,16]. The activation of Wnt/β-catenin with lithium chloride (LiCl) reduces HIV propagation in peripheral mononuclear cells [17], and knockdown of TCF4 or β-catenin enhanced HIV transcription [18].

However, studies on the role of Wnt/β-catenin signaling in influenza virus infection are very limited. In this study, we investigated the effects of activation and inhibition of Wnt/β-catenin signaling on influenza virus replication.

## Results

### Activation of Wnt/β-catenin signaling increases progeny influenza virus production

**Fig 1:**
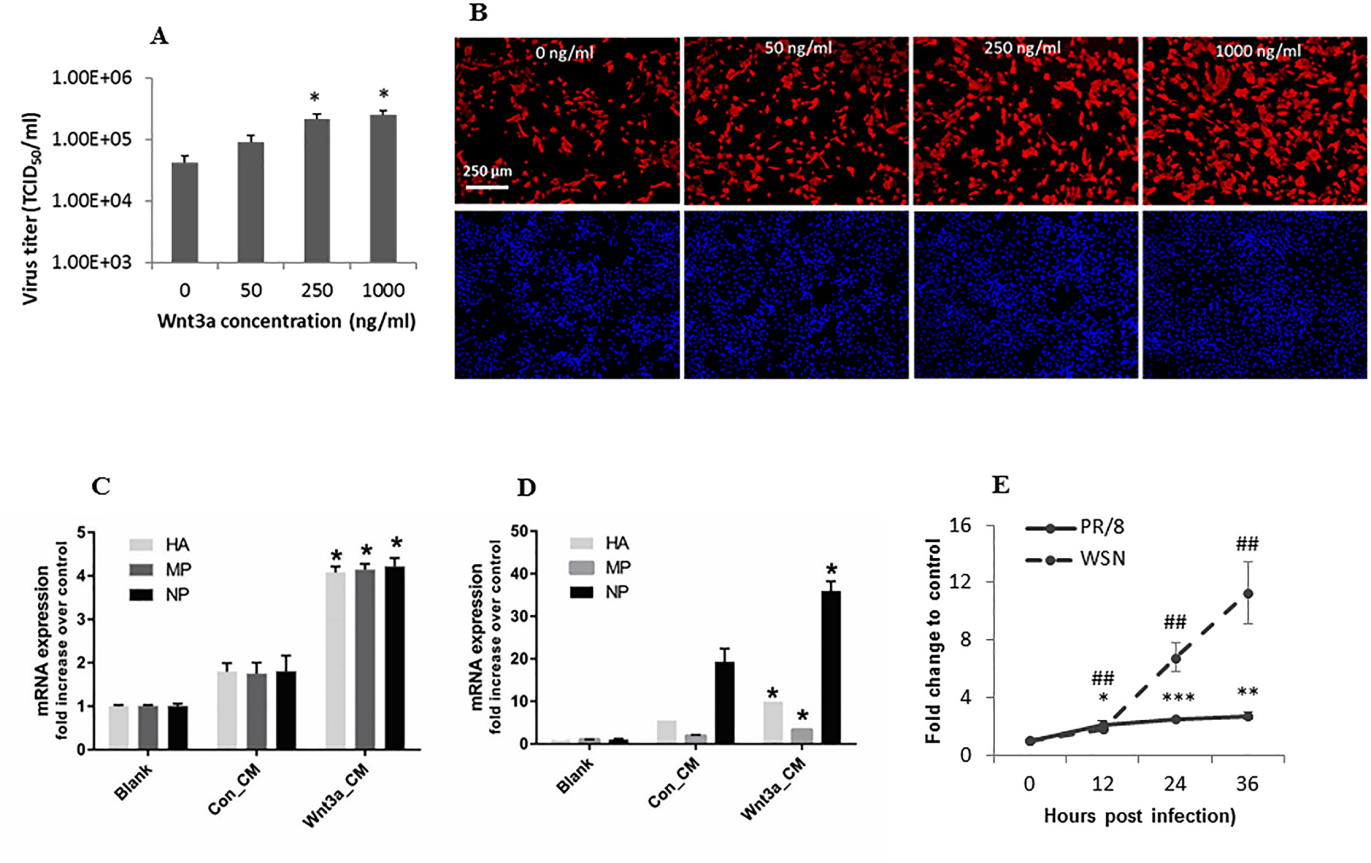
Wnt3a increases progeny influenza virus production. (A, B) Mouse lung epithelial E10 cells were pretreated with different concentrations of recombinant human Wnt3a protein for 24 h and infected with A/PR/8/34 at an MOI of 0.1 for 18 h. The media were collected, and viral titers were determined by TCID_50_ assay. The cells were fixed, and immunostained with anti-NP antibodies. The results of 3 independent experiments are displayed as the mean ± SE. *p<0.05 vs. control (0 ng/ml). (C) E10 cells were pretreated with blank control (DMEM only), 50% Wnt3a_CM or Con_CM for 24 h and infected with influenza virus A/PR/8/34 at an MOI of 1 for 18 h. (D) Mice were instilled with 10x Wnt3a_CM or Con_CM, and the lungs were collected on day 5 post-infection. Relative mRNA expression levels of viral genes (HA, NP and MP) were measured by real-time PCR and normalized to GAPDH. The data are normalized to the blank control and are represented as the mean ± SE (n = 4). *p<0.05 vs. Con_CM. (E) HEK 293 cells were co-transfected with a TOPflash reporter plasmid, and a pRL-TK normalization vector. Twenty four h post transfection, the cells were infected with A/PR/8/34 or A/WSN/33 at a MOI of 0.1 for various times. TOPFlash firefly luciferase activity was normalized to pRL-TK *Renilla* luciferase activity. The results of 4 independent experiments are displayed as the mean ± SE. ##*p*<0.01 vs. control cells without infection (0 time); *p<0.05 vs. control cells without infection; **p<0.01 vs. control cells without infection, ***p<0.001 vs. control cells without infection (Student’s *t-test*).

We first determined the effects of activation of Wnt/β-catenin signaling on progeny virus production and influenza virus gene expression. Mouse E10 lung epithelial cells were pretreated with Wnt3a and then infected with influenza virus A/PR/8/34. The pretreatment with Wnt3a increased influenza virus titer in culture medium (Fig 1A) and NP protein expression as determined by immunostaining (Fig 1B). Wnt3a_CM also increased mRNA expression of the viral genes coding for HA, MP and NP (Fig 1C). When mice were pretreated with Wnt3a_CM and then infected with A/PR/8/34, increases in the mRNA levels of HA, MP and NP in the lungs were also observed (Fig 1D). These results indicate that Wnt3a treatment increased influenza virus replication. Morever, influenza virus infection of HEK293 cells with A/PR/8/34 or A/WSN/33 increased the activities of the Wnt/β-catenin signaling TOPflash reporter although A/WSN/33 were effective (Fig 1E), suggesting that influenza virus activates Wnt/β-catenin signaling.

### Inhibition of Wnt/β-catenin signaling reduces progency influenza virus production

**Fig 2:**
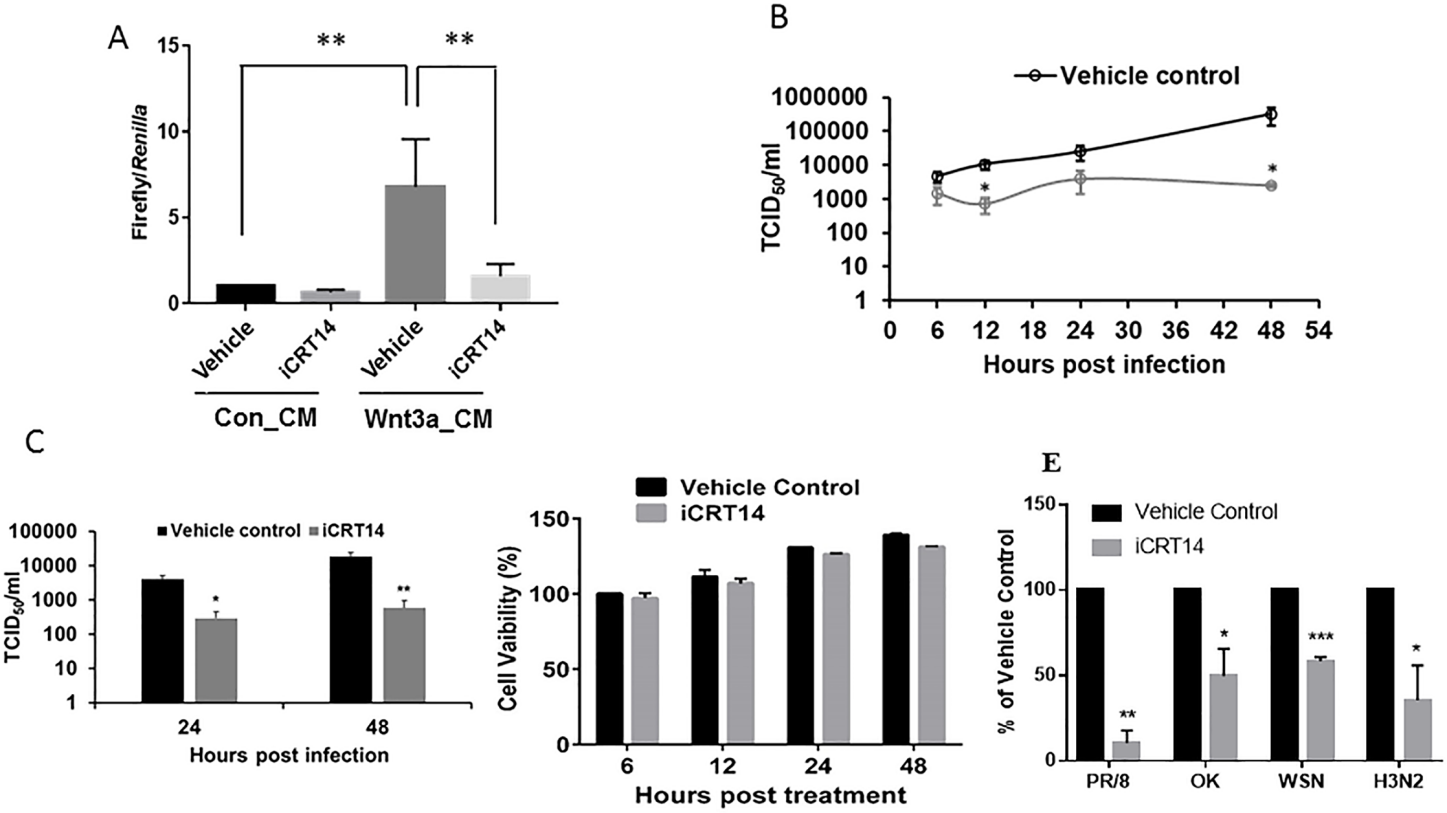
iCRT14 reduces progeny influenza virus production. (A) A549 cells were co-transfected with a TOPflash reporter vector and a pRL-TK normalization vector. Twenty four h post transfection, the cells were treated with Wnt3a_CM or Con_CM in the presence or absence of iCRT14 (12.5 μM) for 24 h. TOPFlash firefly luciferase activity was normalized to pRL-TK *Renilla* luciferase activity. **p<0.01. n = 3 (one-way ANOVA, followed by Tukey's test). (B, C) A549 cells were treated with iCRT14 (12.5 μM) or vehicle control for 12 h and then infected with influenza virus A/PR/8/34 (B) or A/WSN/33 (C) at an MOI of 1. The cell culture medium was collected at various times post-infection, and viral titers were determined by TCID_50_ assay. (D) A549 cells were treated with iCRT14 (12.5 μM) or vehicle control for various times, and cell viability was measured using the CellTiter Glo kit (Promega). The results of 3 independent experiments are displayed as the mean ± SE. *p<0.05 vs. vehicle control, **p<0.01 vs. vehicle control at the corresponding time points (Two-way ANOVA, post hoc Tukey). (E) A549 cells were cotrasnfected with an influenza virus sensor reporter (NP-UTR-Luc) and pRL-TK vector. Twenty-four h post-transfection, the cells were infected with A/PR/8/34, pdm/Ok/09 and H3N2 A/Wisconsin/67/05 at an MOI of 0.1 for 48 h. Firefly luciferase activity was normalized to *Renilla* luciferase activity. *p<0.05 vs. vehicle control, **p<0.01 vs. vehicle control, ***p<0.001 vs. vehicle control (Student’s *t-test*).

We next examined the effect of inhibiting Wnt/β-catenin signaling on influenza virus production. iCRT14, a small molecule that inhibits Wnt/β-catenin signaling by preventing the binding of β-catenin to the LEF/TCF transcription factor [13,19], was used in this study. Using the Wnt/β-catenin signaling TOPflash reporter assay, we first validated that iCRT14 did inhibit the Wnt/β-catenin signaling (Fig 2A). Human alveolar epithelial cells (A549) were then treated with 12.5 μM iCRT14 for 12 h and then infected with influenza virus A/PR/8/34 at a MOI of 1 for varying amounts of time. Infectious virus particles released into the medium were quantified using a TCID_50_ assay. Virus titers in the iCRT14-treated cells were significantly reduced at 48 h post-infection (hpi) compared to the control cells (Fig 2B). Similar results were observed with A/WSN/33 virus (Fig 2C). This effect was not due to the toxicity of iCRT14 because iCRT14 had no effect on cell viability (Fig 2D). We also tested ability of iCRT14 to inhibit virus replication of various influenza virus strains by using an influenza virus sensor reporter assay. As expected, iCRT14 reduced the replication of A/PR/8/34 and A/WNS/33. In this assay, iCRT14 also inhibited the replication of clinical isolates of H1N1 pdm/Ok/2009 and H3N2 A/Wisconsin/67/05 (Fig 2E). These results indicate that iCRT14 has antiviral activity against multiple strains of influenza A virus.

**Fig 3:**
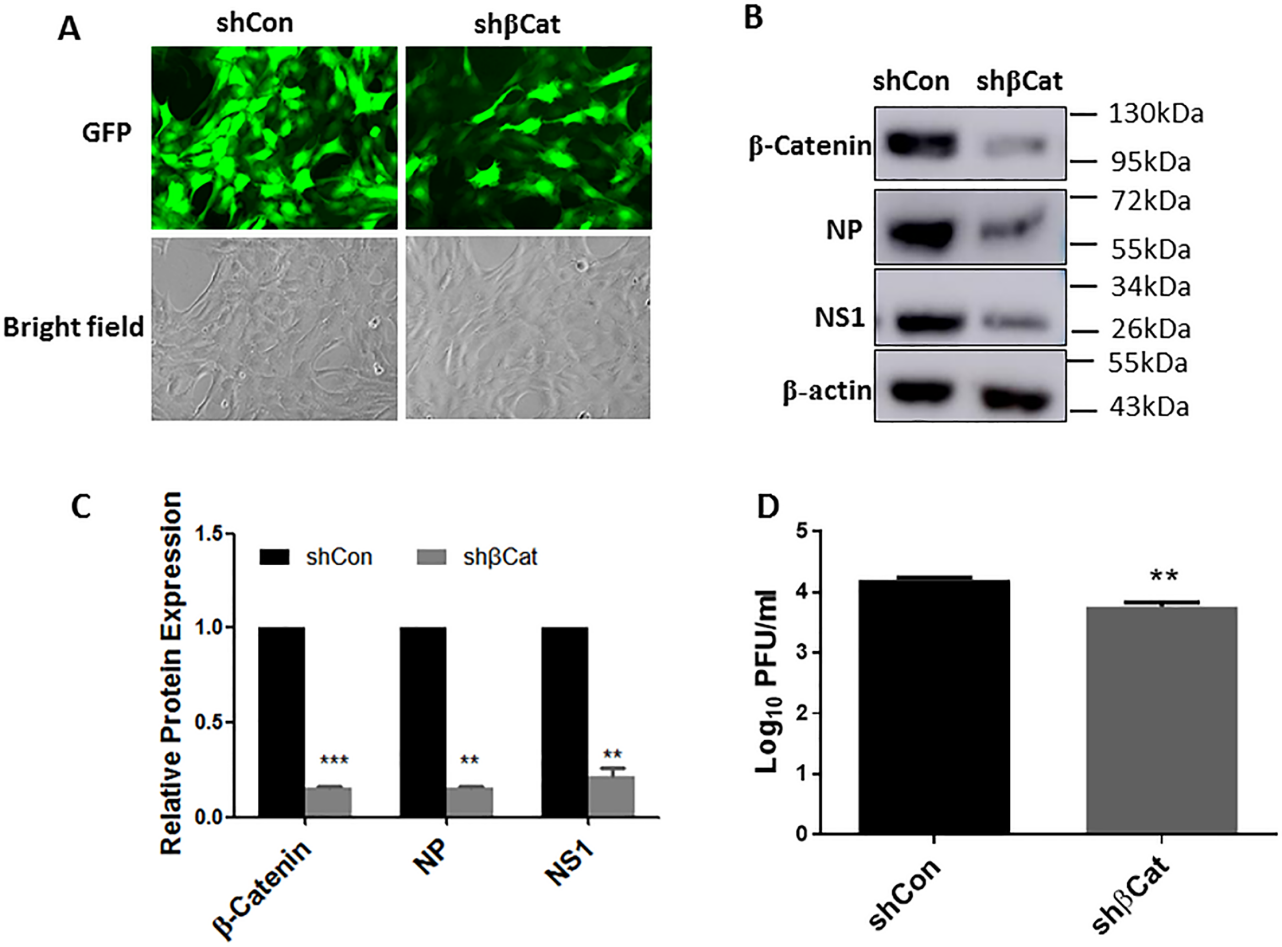
Knockdown of β-catenin reduced viral replication. Rat lung epihtelail L2 cells were treated with adenoviral shRNA targeting β-catenin (shβCat) or a control vector (shCon) at an MOI of 100 for 48 h and then infected with A/PR/8/34 (MOI 0.01) for 48 h. (A) GFP signal after 48 h adenoviral infection. (B) Representaive western blots of β-catenin and viral proteins, NS1 and NP. (C) Quantiation of wesetern blots. (D) Virus titer as determined by plaque assay. **p<0.01 vs. shCon, ***p<0.001 vs. shCon, n = 3 (Student’s *t-test*).

To further confirm the effects of Wnt/β-catenin signaling on influenza virus infection, we inhibited Wnt/β-catenin signaling by knockdown of β-catenin. We have previously shown that knockdown of β-catenin using adenoviral shRNA inhibits the transdifferentitaion of rat alveolar epithelial type II cells to type I cells [20]. Infection of rat lung epithelial L2 cells with the same adenoviral shRNA resulted in a marked reduction of β-catenin expression (Fig 3A–3C). The reduction of β-catenin protein level significantly inhibited influenza viral proteins, NS1 and NP expression (Fig 3B and 3C) and progency influenza virus production (Fig 3D).

### iCRT14 reduces influenza virus RNA synthesis

**Fig 4:**
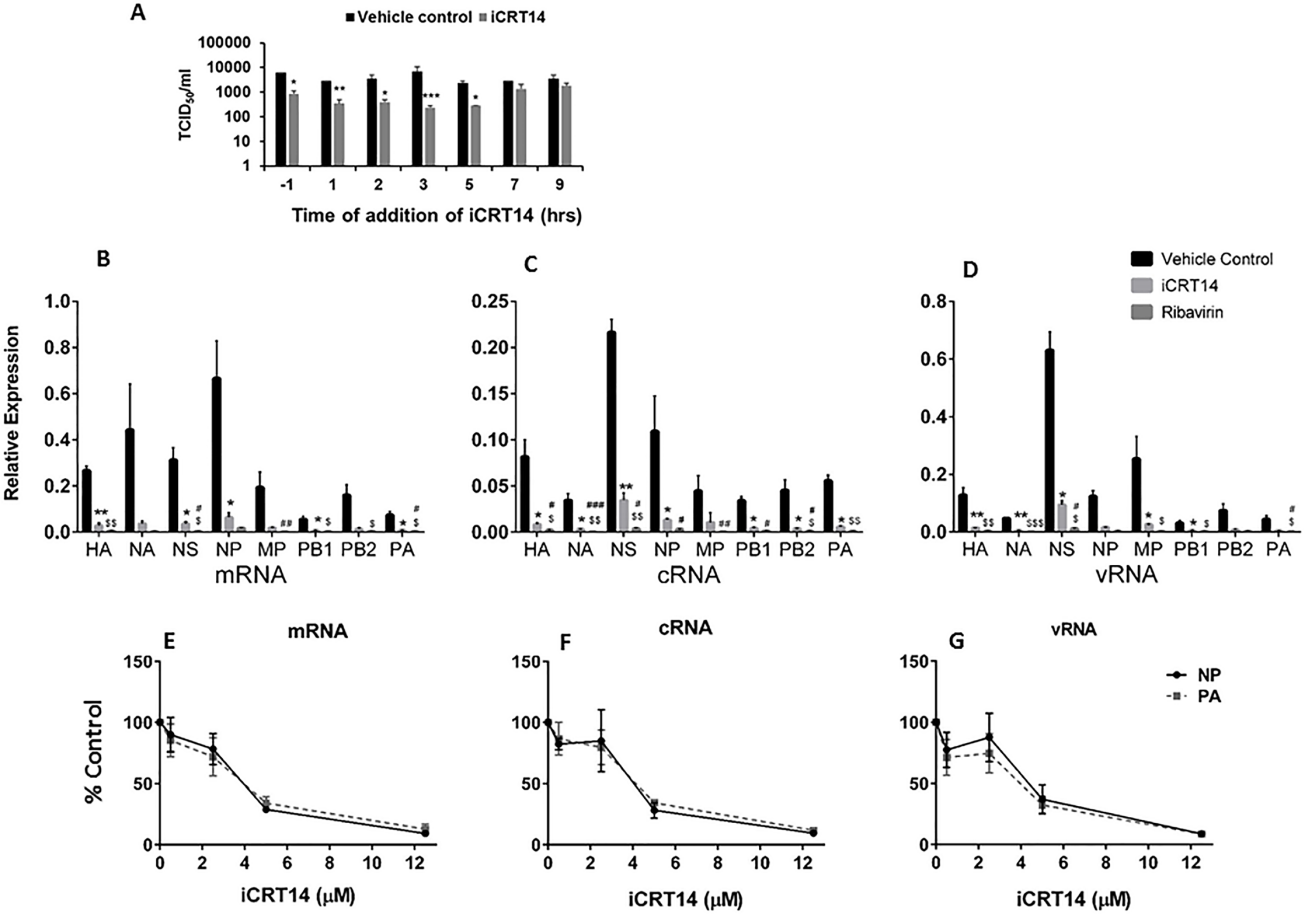
iCRT14 acts during the early stage of the influenza virus life cycle. (A) A549 cells were infected with A/PR/8/34 at an MOI of 1. The cells were treated with iCRT14 (12.5 μM) or vehicle control at various times before or after infection. The culture medium was collected 12 h post-infection, and titers were determined by TCID_50_ assay. The results of 3 independent experiments are displayed as the mean±SE. *p<0.05 vs. vehicle control, **p<0.01 vs. vehicle control, ***p<0.001 vs. vehicle control at the corresponding time points (Two-way ANOVA, post hoc Tukey). (B-G) iCRT14 reduces influenza virus RNA synthesis. A549 cells were infected with A/PR/8/34 at an MOI of 5. The cells were treated with iCRT14 at 12.5 μM (B, C, D) or at the indicated concentrations (E, F, G) 1 h prior to infection. RNA was extracted at 5 h post-infection. The mRNA, cRNA, and vRNA levels of viral genes were determined by real-time PCR and normalized to GAPDH. In B-D, data was expressed as relative expression to GAPDH and in E-G, data was expressed as a percentage of control (without iCRT14). The results of 3 independent experiments are displayed as the mean ± SE. *p<0.05 vs. vehicle control, **p<0.01 vs. vehicle control, ***p<0.001 vs. vehicle control, ^$^*p*<0.05 vs. vehicle control, ^$ $^*p*<0.01 vs. vehicle control, ^$ $ $^*p*<0.001 vs. vehicle control, ^#^*p*<0.05 vs. iCRT14, ^##^*p*<0.01 vs. iCRT14, ^###^*p*<0.001 vs. iCRT14 (One-way ANOVA, post hoc Tukey).

Influenza virus undergoes several steps of its life cycle in the host cell during the period from entry to budding [21]. To determine which stage of the viral life cycle is inhibited by iCRT14, we added the inhibitor at various times before and after infection and determined the virus titer in the medium at 12 hpi. When iCRT14 was added 1 h before or up to 5 hpi, virus titer decreased (Fig 4A). However, iCRT14 had no effect on virus titer when it was added at 7 or 9 hpi. The results indicate that iCRT14 acts at the early stages of viral infection at or before viral gene transcription.

We further examined the effect of iCRT14 on the expression levels of various influenza viral RNAs. A549 cells were treated with iCRT14 (12.5 μM) 1 h prior to infection and infected with influenza virus A/PR/8/34 at a MOI of 5 for 5 h; cRNA, vRNA, and mRNA were then measured by real-time PCR. Ribavirin, which inhibits influenza virus RNA synthesis by inhibiting the polymerase complex [22], was used as a positive control for this experiment. Similar to ribavirin, iCRT14 markedly reduced the levels of vRNA, cRNA and mRNA of all 8 segments of viral RNA, including polymerase basic 1 (PB1), polymerase basic 2 (PB2), polymerase acidic (PA), HA, NP, NA, MP and non-structural protein (NS) (Fig 4B–4D). This effect was dose-dependent (Fig 4E–4G). The results show that iCRT14 inhibits viral gene replication and transcription.

### Interferons and antiviral activity of iCRT14

**Fig 5:**
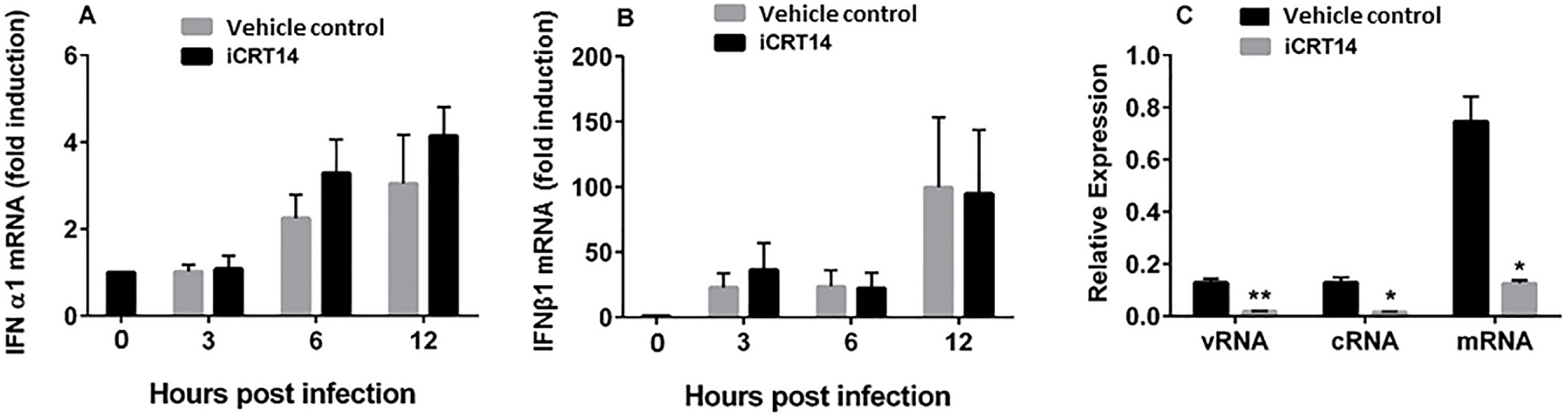
Effects of iCRT14 on influenza virus-induced IFN response and iCRT14-mediated inhibition of influenza virus RNA synthesis in Vero cells. (A, B) A549 cells were infected with A/PR/8/34 at an MOI of 1 for various times. The cells were treated with iCRT14 (12.5 μM) or vehicle control 1 h prior to infection. IFN α1 and β1 mRNA levels were determined by real-time PCR and normalized to GAPDH. The data are expressed as fold change relative to mock infection. (C) Vero cells were infected with A/PR/8/34 at an MOI of 5. The cells were treated with iCRT14 (12.5 μM) or vehicle control 1 h prior to infection. The cRNA, vRNA and mRNA levels of the NP gene were determined at 5 h post-infection using real-time PCR and normalized to GAPDH. The data are expressed as relative expression. The results of 3 independent experiments are presented as the mean ± SE. *p<0.05 vs. vehicle control, *p<0.01 vs. vehicle control (Student’s *t-test*).

Interferons (IFNs) are the first line of defense against influenza virus. Influenza virus infection induces IFN production, which is required for mounting a proper antiviral response [23]. We designed two sets of experiments to test whether IFNs are involved in the antiviral activity of iCRT14. First, we examined the effects of iCRT14 on influenza virus A/PR/8/34-induced IFN gene expression. A/PR/8/34 induced the mRNA expression of IFNα1 and IFNβ1 in A549 cells. However, iCRT14 had no effect on this induction (Fig 5A and 5B). Second, we repeated the experiment described in Fig 4 using Vero cells, which are deficient in IFN production [24,25]. iCRT14 reduced the levels of vRNA, cRNA and mRNA of the NP gene in Vero cells, similar to its effect in A549 cells (Fig 5C). These results suggest that IFNs may not play a major role in the antiviral activity of iCRT14.

### iCRT14 reduces influenza virus infection in primary alveolar epithelial cells

**Fig 6:**
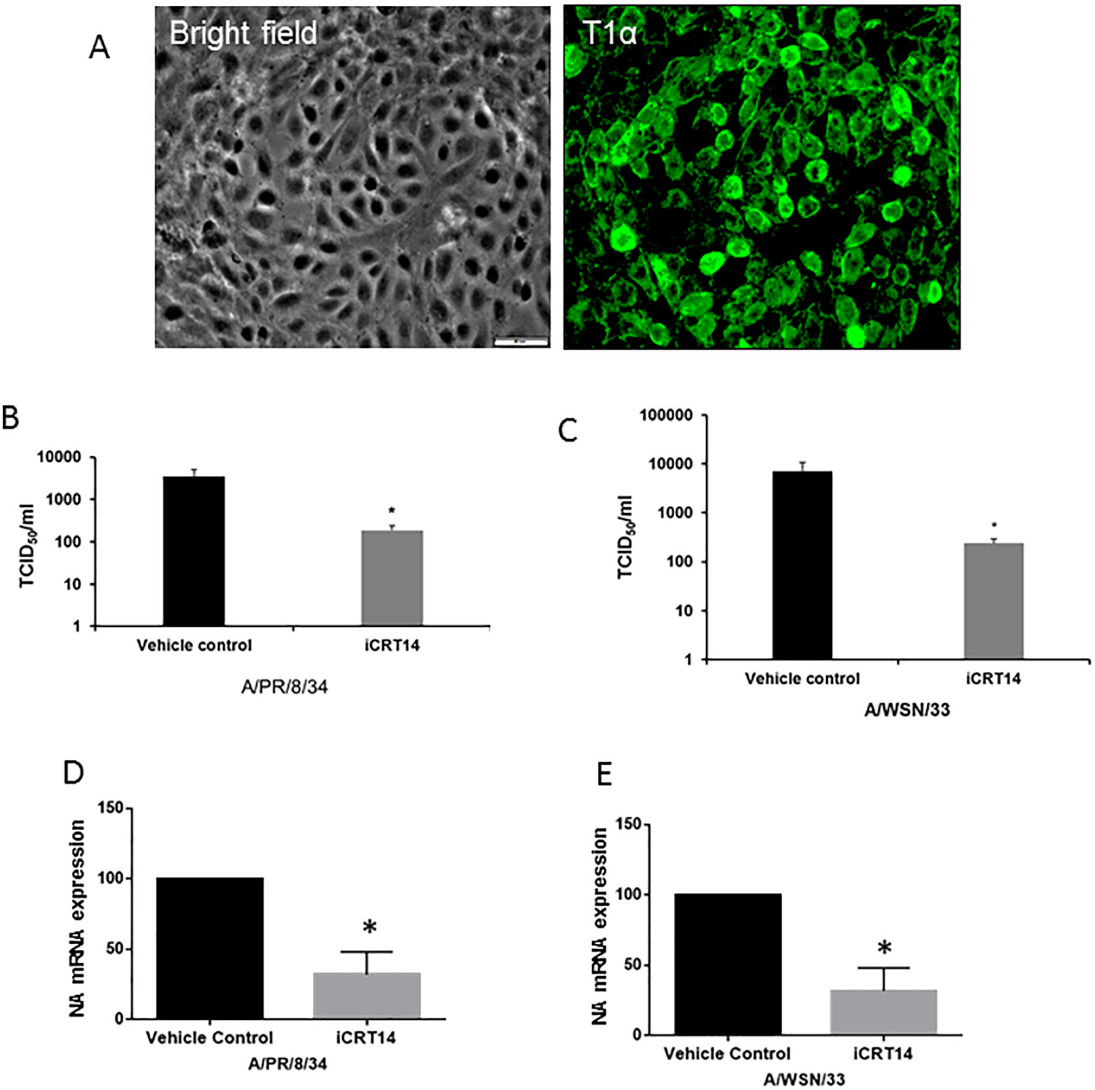
iCRT14 reduces influenza virus infection in primary mouse alveolar epithelial cells (AECs). Mouse AECs were isolated and cultured for 6 days. (A) AECs were immuno-stained with the AEC type I marker T1α and the nuclear dye DAPI. Scale bar = 50 μm. (B-E) AECs were infected with influenza virus A/PR/8/34 or A/WSN/33 at an MOI of 1 for 24 h. The cells were treated with iCRT14 (12.5 μM) or vehicle control 1 h prior to infection. Viral titers in the cell culture medium were determined using the TCID_50_ assay (B, C). NA mRNA levels in cells were determined by real-time PCR and normalized to GAPDH. The results of 3 independent experiments are displayed as the mean ± SE. *p<0.05 vs. vehicle control at the corresponding time points (Student’s *t-test*).

Alveolar epithelial cells (AEC) are one of the primary targets of influenza virus. We examined whether iCRT14 also reduces influenza virus infection in primary AEC. AEC II were isolated from mice and cultured for 6 days to allow them to differentiate into AEC I [26,27]. On day 6, the cells showed uniform staining of the AEC I marker T1α (Fig 6A). We treated these cells with iCRT14 or with vehicle, infected them with influenza virus A/PR/8/34 or A/WSN/33 at a MOI of 1 for 24 h, and measured virus titers in the medium and NA mRNA in the cells. We found that iCRT14 reduced virus titers (Fig 6B and 6C) and NA mRNA levels (Fig 6D and 6E) in both A/PR/8/34- and A/WSN/33-infected cells. Our results indicate that iCRT14 is also effective in repressing influenza virus production in primary AEC.

### iCRT14 toxicity

We evaluated whether iCRT14 causes toxicity in mice before testing antiviral activity of iCRT14 *in vivo*. Mice were treated with iCRT14 at doses of 50 and 100 mg/kg or vehicle control daily for 9 days via the intraperitoneal route and sacrificed at day 10. Major organs, including the liver, lung, heart, kidney, stomach, small intestine, eyes and brain, were processed for histopathological analysis. Microscopic lesions were scored by a pathologist. Major organ toxicity after iCRT14 treatment was not observed at either dose of iCRT14.

### iCRT14 partially protects mice from challenge with a lethal dose of influenza virus

**Fig 7:**
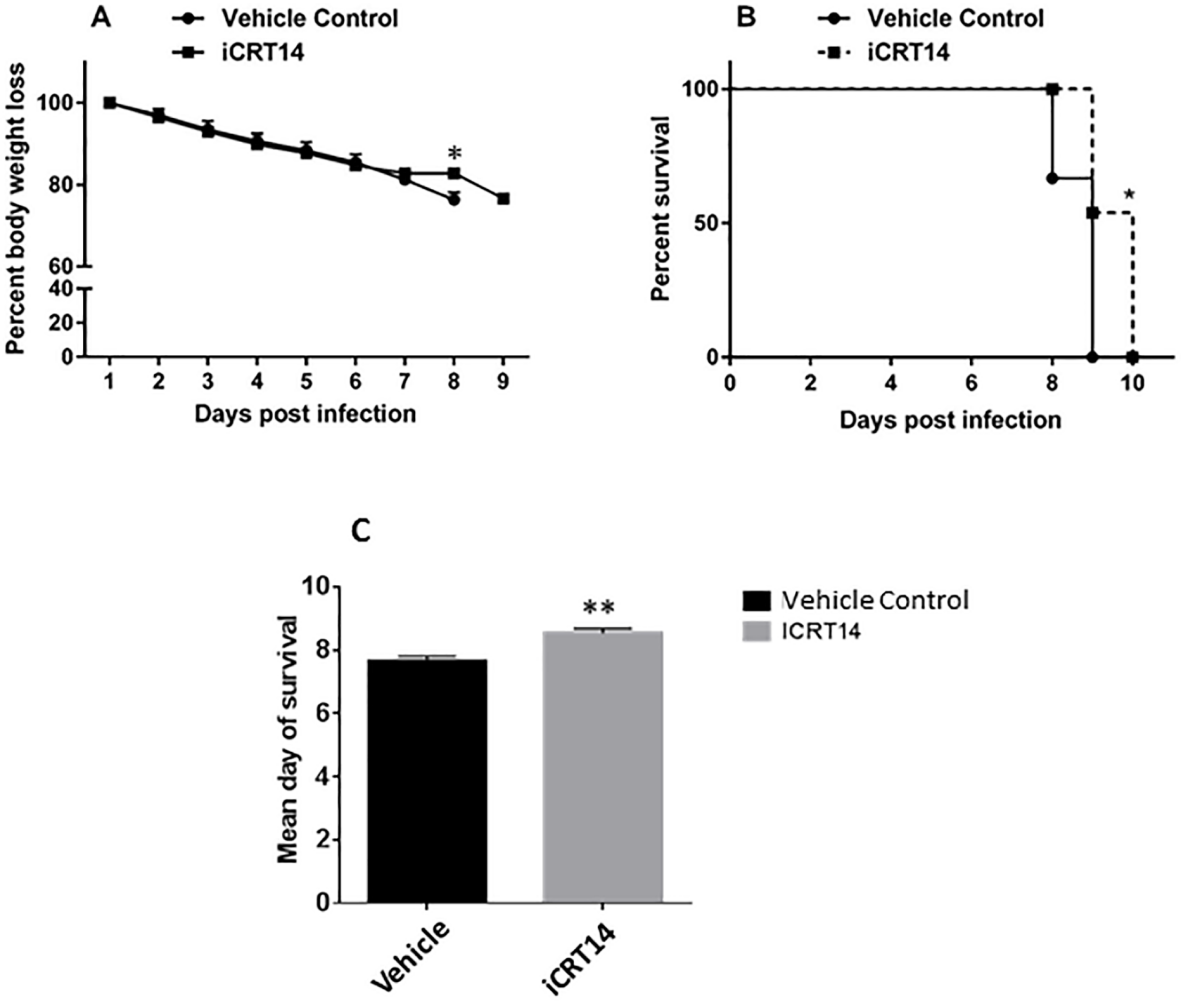
Effects of iCRT14 on weight loss and survival rate in mice challenged with a lethal dose of influenza virus A/PR/8/34. C57BL/6J mice were treated with iCRT14 (50 mg/kg) or vehicle control daily from one day prior to infection until day 6 post-infection. The mice were challenged with influenza virus A/PR/8/34 (1,000 pfu/mouse). (A) Percent of body weight loss. Body weight loss is presented as the mean ± SE. *p < 0.05 vs. vehicle control at day 8 (Two-way ANOVA followed by post-hoc sidak test). (B, C) Kaplan–Meier survival curves and mean day of survival of mice in the iCRT14 and vehicle control groups. The Mantel-Cox χ^2^ test and Student’s t-test were used for survival curves and mean day of survival, respectively. *p< 0.05 vs. control group; **p<0.01 v.s control group, n = 12–13 animals per group.

To test whether iCRT14 protects mice from influenza virus infection, we performed a survival study using a lethal dose of influenza virus A/PR/8/34 (1,000 pfu/mouse). Twenty-five mice were treated with iCRT14 (50 mg/kg body weight) or vehicle (DMSO, 4.2%) one day prior to infection and daily thereafter until day 6 after infection. The dose was chosen based on published reports of tumor studies [13,28]. Animal survival was monitored daily after IAV infection. From a total of 25 mice (iCRT14-treated, vehicle control), 0 mice survived, 5 mice died before reaching 30% body weight loss, and 20 mice were euthanized according to the criteria for euthanasia. Though all mice died within 10 days after infection, the iCRT14-treated mice exhibited slightly less body weight loss at day 8 (Fig 7A) and a slight improvement in survival time (Fig 7B and 7C).

### iCRT14 attenuates the clinical signs associated with challenge with a sublethal dose of influenza virus

**Fig 8:**
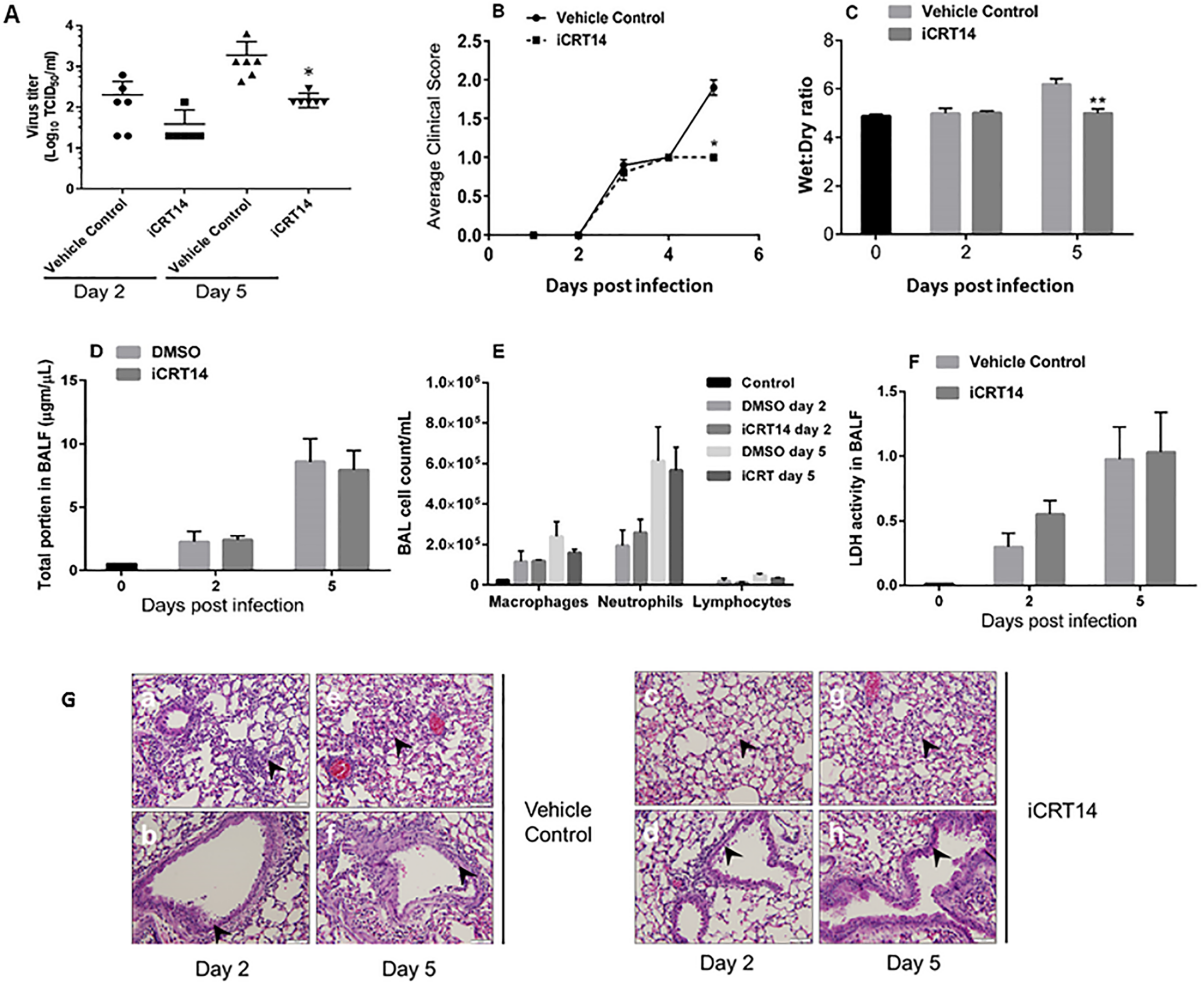
Effect of iCRT14 on lung injury and inflammation of mice challenged with a sublethal dose of influenza virus A/PR/8/34. C57BL/6J mice were infected with A/PR/8/34 (100 pfu/mouse). Mice euthanized on day 0 of the study were used as a control. Mice received either iCRT14 (50 mg/kg) or vehicle control daily beginning one day prior to infection until day 2 or day 5 after infection. (A) Virus titers in total lung homogenate (n = 6). (B) Average clinical scores; 0 = normal, 1 = ruffled fur, 2 = inactive, 3 = hunched back (n = 10–20). (C) Lung water content as measured by wet-to-dry ratio (n = 4). (D) Total protein in BAL fluid (n = 4). (E) LDH activity in BAL fluid (n = 4). (F) Inflammatory cells in BAL (n = 3–4). The data are presented as the mean ± SE. *p < 0.05 vs. vehicle control on the corresponding days, **p < 0.01 vs. vehicle control on the corresponding days. For (A) and (C), Two-way ANOVA, post hoc sidak was used and 0 time group was not included in analysis due to lack of equal groups. For (B), Student’s t-test was used duo to unequal animal numbers in different groups. (G) Representative H&E photomicrographs from 6 mice are shown. A, B = day 2 vehicle control mice; C, D = day 2 iCRT14 mice; E, F = day 5 vehicle control mice; G, H = day 5 iCRT14 mice. The dark arrowheads in Panels A, C, E, G represent alveolar histiocytic and neutrophil infiltrations with associated hemorrhage. The arrowheads in Panels B, D, F, H represent major airway degeneration/necrosis, histiocytic and neutrophil infiltration. Scale bar = 50 μm.

We challenged mice with a sublethal dose of A/PR/8/34 (100 pfu/mouse) and examined the resulting virus loads and lung pathology. Body weight and animal survial were observed for 5 days. In a total of 40 mice, no mortality was observed in either the control or the iCRT14-treated group. There was also no difference in body weight loss between the control and the iCRT14-treated groups. iCRT14 significantly reduced virus titers in the lung tissues at 5 days’ post-infection (Fig 8A). iCRT14-treated mice showed significantly lower clinical scores than control mice at day 5 post-infection (Fig 8B). The mice treated with iCRT14 displayed a decreased wet-to-dry ratio, an indicator of extravascular edema in the lungs, at 5 days after infection (Fig 8C). We did not observe differences in total protein, number of inflammatory cells (macrophages, neutrophils and lymphocytes) or LDH activity in BAL from the iCRT14-treated and control groups (Fig 8D–8F), suggesting that iCRT14 does not improve alveolar leakage, cell injury or inflammation.

H&E staining revealed that at day 2 post-infection iCRT14 equivocally reduced epithelial degeneration and/or necrosis in major airways compared to vehicle control (Table 1). At day 5 post-infection, there was decreased attenuation of epithelium and loss of cilia in the lungs of animals that received iCRT14 treatment. There was a subtle decrease in the severity of the lung lesions, such as alveolar histiocytic infiltration and neutrophilic and histiocytic infiltration in major airways, in the treatment group at day 5 post-infection compared to the control group (Fig 8G, Table 1).

**Table 1:** Histopathology of the lungs of mice on days 2 and 5 after infection with a sublethal dose of influenza virus.

Microscopic Findings in Lung	Treatment Groups											
	Vehicle control						iCRT14 (50 mg/kg)					
Animal #	7	8	9	10	11	12	13	14	15	16	17	18
**2 days post-infection**												
Major airways: Epithelial degeneration and/or necrosis	3M	--	3M	3M	--	2M	2M	2M	2M	2M	3M	2M
**5 days post-infection**												
Animal #	19	20	21	22	23	24	25	26	27	28	29	30
Alveolus: Histiocyte infiltration	3M	3M	3M	3M	3M	2M	3M	2M	2M	1M	3M	3M
Major airways: Attenuated epithelium with loss of cilia	3M	2M	3M	3M	3M	2M	2M	1M	1M	2M	2M	2M
Major airways: Neutrophil and histiocyte infiltration	3M	2M	3M	3M	2M	2M	3M	2M	1M	1M	2M	3M

Severity: -- = not present; 1 = minimal; 2 = mild; 3 = moderate Distribution: F = focal; M = multifocal

## Discussion

In this study, we found that activation of Wnt/β-catenin with Wnt3a enhanced influenza virus replication and that inhibition of this pathway with iCRT14 decreased influenza virus transcription and the production of new virus. iCRT14 acted at or before viral RNA synthesis. iCRT14 also showed a partial protective effect in a mouse model of influenza virus infection.

Various host signaling pathways are altered during influenza virus infection; thus, these pathways might provide potential therapeutic targets. Cellular signaling pathways such as nuclear factor kappa-light-chain-enhancer of activated B cells (NF-κB) signaling, the mitogen-activated protein kinases (MAPK) pathway, the phosphoinositide 3-kinase (PI3K/Akt) pathway, Protein kinase RNA-activated (PKR) signaling, and toll-like receptor/retinoic acid-inducible gene 1 (TLR/RIG-I) signaling cascades have been reported to play roles in various stages of the influenza virus replication cycle. NF-κB signaling is essential for the host innate immune response and is activated by influenza virus infection [29]. Once activated, NF-κB signaling increases influenza virus production by inducing proapoptotic factors [30]. Influenza virus also activates the MAPK pathway, and blocking this signaling pathway inhibits viral replication by impairing viral ribonucleoprotein trafficking [31]. Another pathway, the PI3K/Akt pathway, is activated by influenza virus via the interaction of non-structural 1 (NS1) protein and p85β [32]. During influenza virus infection, robust activation of the IFN response occurs, and this in turn activates the PKR signaling pathway. However, influenza virus also counteracts activation of the PKR pathway by the binding of NS1 to double-stranded RNA, thereby preventing translational arrest [33]. Pattern recognition receptors such as TLRs and RIG-I like receptors (RLRs) recognize viral RNAs and induce antiviral immune responses [34]. Influenza virus has also evolved mechanisms to counteract this induction of the antiviral response. For example, NS1 inhibits RIG-I activation and IFN production by binding to tripartite motif-containing protein 25 (TRIM25), an ubiquitin ligase that is required for RIG-I activation [35].

The current study demonstrates that the activation of Wnt/β-catenin signaling enhances influenza virus replication. We showed that Wnt3a enhances viral gene expression in H1N1 A/PR/8/34-infected mouse lung epithelial cells in cell culture and in H1N1 A/PR/8/34-infected lungs *in vivo*. We also demonstrated that the Wnt/β-catenin inhibitor iCRT14 inhibits virus replication and viral gene expression in H1N1 A/PR/8/34- and A/WSN/33-infected human lung epithelial A549 cells and primary mouse alveolar epithelial cells. This effect was also observed with clinical isolates of H1N1 pdm/Ok/2009 and H3N2 A/Wisconsin/67/05.

There are limited studies on the effects of Wnt/β-catenin signaling on influenza virus infection. A yeast two-hybrid screening reveals that viral proteins including NS1, PB1, PB2, NP and PA interact with Wnt signaling componments [10]. Using an RNAi approach, silencing of various components in Wnt/β-catenin signaling affects A/PR/8/34 replication. However, the results of this study are difficult to interpret because deletion of these genes can have either a positive or a negative effect on A/PR/8/34 replication. However, deletion of FzD5, DAAM2 and CSNK1A1L reduced virus replication [10], consistent with our inhibitor studies. Another study showed that addition of Wnt3a or overexpression of β-catenin and LEF1 inhibits H7N7 influenza virus replication [36]; this contrasts with our observation that Wnt3a actually enhanced H1N1 viral replication and knockdown of β-catenin reduces H1N1 influenza virus replication. The discrepancy is likely due to differences in the strains used.

The mechanisms how Wnt/β-catenin signaling regulates influenza virus infection are unclear. There are several possibilities: (i) Modulation of immune response. Wnt/β-catenin signaling pathway is known to modulate inflammatory and immune responses via the interaction with NF-κB pathway [37]. However, the effect of Wnt/β-catenin signaling on virus-induced immune responses remains controversial. For instance, Hillesheim *et al* has shown that activation of Wnt/ β-catenin signaling pathway by overexpressing β-catenin supports the IAV-induced IRF3-dependent transcription of genes of cellular innate immune response, such as the IFN-β and ISGs [36]. However, a recent study has demonstrated that induction of Wnt ligands by negative-strand RNA virus infection suppresses the type I IFN response. Our current studies do not support a role of type I IFN response in this context as the inhibitory effect of the Wnt/β-catenin inhibitor iCRT14 on influenza virus infection was not via the enhancement of IFN response (Fig 5). (ii) Modulation of cell cycle and proliferation. Wnt/β-catenin signaling promotes the G1/S transition through transcriptional upregulation of cell cycle related target genes such as c-Myc and cyclin D [38]. A proliferative status promoted by Wnt/β-catenin signaling may: 1) provide abundant resource for virus cap snatching. This has been shown for another negative strand RNA virus, Rift Valley Fever Virus, which selectively snatches cell cycle-related cellular mRNAs [39]; 2) allow the viral protein traffic between cytoplasm and nucleus more actively. For instance, Cawood et al has demonstrated that the nucleocapsid protein of Coronavirus is more mobile in proliferative cells and localizes to the nucleolus in a cell cycle-dependent manner [40].

We also showed that iCRT14 maintains its ability to inhibit influenza virus replication in IFN-deficient Vero cells and that it has no effect on the influenza virus-induced type I IFN gene expression in A549 cells. Different effects of Wnt/β-catenin on IFN response during virus infection have been reported. The Wnt ligands Wnt2b and Wnt9b negatively regulate the IFN response during Sendai virus infection [41], and deletion of Wnt9b positively regulates the IFN response [10]. In contrast, Wnt3a increases the IFN response in the absence of NS1 protein, the main protein that influenza virus uses to repress the host IFN response [10]. Transfection of β-catenin and LEF-1 plasmids into A549 cells also induces the interferon-sensitive response element (ISRE) gene reporter when the cells are treated with cellular or viral RNA [42].

In a xenograft model of BT-474 cells in severe combined immunodeficiency (SCID) mice, iCRT14 treatment was shown to significantly reduce tumor volume [28]. Mouse Ewing’s sarcoma *in vivo* was also inhibited by iCRT14 [43]. However, there is a paucity of literature on the toxicity of iCRT14 in animals. Our 10-day toxicity study in mice did not reveal any major toxicity to organs, including lung, liver, kidney, brain, heart and eyes. In a sublethal influenza infection mouse model, iCRT14-treated mice showed less severe clinical signs, and this correlated with the observation that those mice had lower virus loads at 5 days post infection. Virus load was less in the iCRT14-treated group at 2 days post infection, however it did not reach a significant level. Although we believe this is due to lower titer at day 2 and animal variations, we can not rule out the possibility that iCRT14 eexerts secondary effects in animals. Influenza virus infection causes acute lung injury, including damage to endothelial and epithelial cells, disruption of the endothelial alveolar barrier, leakage of proteins into the alveolar space, and inflammatory cell infiltration. However, iCRT14 appears to have no effects on parameters of lung injury and inflammation other than a reduction in lung edema. In a lethal influenza infection, we observed only slightly less weight loss and slightly delayed mortality in iCRT14-treated mice. These end points could be used to improve the efficacy of iCRT14 using medicinal chemistry and formulation. Because iCRT14 reduced virus loads and improved clinical signs, combination therapy [44] with other drugs that reduce lung injury should increase the efficacy of iCRT14.

## Conclusions

The present study provides evidence that the activation of Wnt/β-catenin signaling increases influenza virus gene expression and virus production and the inhibition of Wnt/β-catenin signaling limits influenza virus production by reducing viral RNA synthesis.

## Materials and methods

### Cell culture

A549 (human lung epithelial) and MDCK (Madin-Darby canine kidney epithelial) cells were purchased from the American Type Culture Collection (ATCC, Manassas, VA, USA). A549 cells were maintained in F12K medium with 10% fetal bovine serum (FBS) and 1% penicillin and streptomycin (PS). MDCK cells were maintained in DMEM:F12K (1:1; volume/volume) medium with 10% FBS, 1% PS and 1% insulin-transferrin-sodium selenite medium supplement (ITS) (Corning, NY, USA). Vero (African green monkey kidney epithelial) cells (ATCC) were maintained in DMEM with 10% FBS and 1% PS. E10 cells, a lung epithelial cell line, were kindly provided by Dr. M. Williams (Boston University) and were maintained in CMRL (Connaught Medical Research Laboratories) medium with 10% FBS, 1% PS and 2.5 mM L-Glutamax^®^.

### Isolation of mouse primary epithelial cells

Mouse alveolar epithelial cells type II (AEC II) were isolated from male C57BL/6 mice (8–10 weeks of age) as previously described [26]. All experiments were carried out with the approval of the Oklahoma State University Institutional Animal Care and Use Committee in accordance with the Public Health Service (PHS) Policy on Humane Care and Use of Laboratory Animals. Mice were anesthetized with ketamine and xylazine. The abdominal cavity was opened, the animals were exsanguinated by interrupting the abdominal aorta, and the lungs were cannulated with a 20-gauge catheter via the trachea. The lungs were perfused with solution II (10 mM HEPES, pH 7.4, 0.9% NaCl, 0.1% glucose, 5 mM KCl, 1.3 mM MgSO_4_, 1.7 mM CaCl_2_, 0.1 mg/ml streptomycin sulfate, 0.06 mg/ml penicillin G, 3 mM Na_2_HPO_4_ and 3 mM NaH_2_PO_4_), followed by instillation of 1 ml of solution I (15 ml solution II plus 10 ml dispase from a stock of 50 caseinolytic units/ml) through the trachea. Three lungs were isolated, pooled in a beaker containing approximately 10 ml of solution I and incubated at 37°C for 45 min to release the AEC. After incubation, the lungs were chopped and further digested by the addition of DNase I (100 μg/ml) to solution I (10 ml) for 45 min at 37°C with intermittent shaking. The digested lungs were filtered sequentially through 160-, 37- and 15-μm gauge nylon mesh. The filtrate was centrifuged at 250 *g* for 10 min. The cell pellet was resuspended in DMEM and incubated in a 100-mm-diameter Petri dish coated with mouse IgG (75 μg per dish) for 1 h. The cells were centrifuged at 250 *g* for 10 min and resuspended in DMEM containing 10% FBS. The yield was approximately 10^7^ cells per 3 mice, and the cell viability was >95%.

### Preparation of Wnt3a conditioned medium

A stable cell line expressing soluble murine Wnt3a and a control murine L-cell line (ATCC) were maintained in DMEM supplemented with 10% FBS, 1% L-glutamine, and 0.4 mg/ml G418 (Invitrogen, Carlsbad, CA, USA). To obtain Wnt3a_, or control (Con)_conditioned medium (CM), cells were cultured in fresh growth medium (DMEM with 10% FBS) without G418 for 4 days and then in fresh G418-free medium for an additional 3 days. The cultured media were mixed, sterile filtered, and stored at −80°C until use. 10X Wnt3a_CM and Con_CM were prepared by concentrating 20 ml CM to 2 ml using an ultrafiltration kit (Millipore, Billerica, MA, USA).

### Viruses

Stocks of H1N1 strains of influenza virus A/PuertoRico/8/34 (A/PR/8/34) and A/WSN/1933 (A/WSN/33) as well as clinical isolates of H1N1 A/Oklahoma/3052/2009 (pdm/Ok/09) and H3N2 A/Wisconsin/67/05X-161B (A/Wisconsin/67/05) were propagated in the allantoic cavities of 10-day specific-pathogen-free embryonated chicken eggs (Charles River Laboratories, MA, USA) at 35°C. The allantoic fluid was harvested, centrifuged at 2,000 *g* for 10 min, and stored at −80°C. Virus titer was determined by a Tissue Culture Infective Dose (TCID_50_) assay. Briefly, MDCK cells were seeded in 96-well plates at a density of 25,000 cells per well. The next day, the cells were washed twice with serum-free medium. A series of ten-fold dilutions of stocks of allantoic fluid or cell culture supernatant ranging from 10^−1^ to 10^−8^ was prepared in serum-free medium with 2 μg/ml L-1-tosylamide-2-phenylethyl chloromethyl ketone-treated trypsin (TPCK-trypsin). The cells were infected with each diluted virus stock in triplicate. After 72 h of culture, the cells were analyzed for cytopathic effect (CPE), and TCID_50_ was calculated using the Reed-Muench method [45].

### Virus infection

Primary AEC II cells were cultured in type I collagen-coated 12-well plates at 0.25×10^6^ cells/well or on cover slips for 6 days. All other cells were seeded at a density of 0.5×10^6^ cells/well in 6-well plates for 24 h. The cells were washed twice with serum-free medium. The cells were infected with A/PR/8/34 or A/WSN/33 virus in serum-free medium containing TPCK-trypsin (2 μg/ml) at the desired multiplicity of infection (MOI) at 37°C in a 5% CO_2_ incubator for 1 h. The cells were then washed once with PBS, and complete medium containing 10% FBS and 1% PS was added. At 24 h post-infection, total viral mRNA in the cells and virus titer in the medium were determined.

### Knockdwon of β-catenin

Rat lung epithelial L2 cells (ATCC) were seeded in a 12-well plate (2 x 10^5^/well) and cultured in F12K medium containing 10% FBS and 1% PS for 24 h. Cells were infected with an adenovirus containing β-catenin shRNAs or a control vector at a MOI of 100 as previously described [20]. 24 h after infection, adenovirus was removed. Culture medium was then added. After culture for another 24 h, cells were washed with serum-free media and infected with A/PR/8/34 PR8 (MOI, 0.01) for 1 h in serum-free medium containing 0.5 μg/ml TPCK-trypsin. Influenza virus was then removed and cells were cultured in serum-free medium containing 0.3% BSA and 0.5 μg/ml TPCK-trypsin for 48 h. Cells were lysed for western blotting and media were used for plague assay. The primary antibodies used include rabbit anti-β-catenin (Catalog No, 9562S, lot#13, Cell Singalling, Becerly, MA, 1:1000 dilution), mouse anti-NP (Catalog No, HB-65, ATCC, 1:40 dilution), mouse anti-NS1 (Catalog No, sc-130568, lot#D0517, Santa Cruz Biotechnology, California, CA, 1:1000 dilution), mouse anti-β-actin (Catalog No, MA5-15739-1MG, lot#QK229411, Sigma, St Louis, MO, 1:3000 dilution). Secondary Antibodies include peroxidase-conjugated goat anti-mouse IgG (H+L) (Catalog No.115-035-003, lot#89918, Jackson Immuno Research, West Grove, PA, 1:2000 dilution), and peroxidase-conjugated goat anti-rabbit IgG (H+L) (Catalog No.111-035-003, lot#130223, Jackson Immuno Research, West Grove, PA, 1:2000 dilution).

### Immunofluorescence

Alveolar epithelial cells were fixed with ice-cold 4% paraformaldehyde for 20 min and washed with PBS. The cells were permeabalized with 0.1% Triton X-100 for 10 min, washed again, blocked with 5% goat serum and incubated with hamster anti-T1α antibody (1:100 dilutions; E11, Developmental Studies Hybridoma Bank, University of Iowa) at 4°C overnight followed by incubation with an Alexa Fluor 488-conjugated anti-hamster secondary antibody (Life Technologies, Grand Island, NY, USA) at 1:500 dilutions. Finally, the cells were stained with 4’,6-diamidino-2-phenylindole dihydrochloride (DAPI). Coverslips were mounted on glass slides for imaging.

### Cell viability assay

iCRT14 (Tocris Bioscience, Minneapolis, MN, USA) stock solution (25 mM) was prepared in dimethyl sulfoxide (DMSO) and diluted to yield the desired concentration in the appropriate medium for different cell types. DMSO (0.05%) was used as a vehicle control. For the cell viability assay, A549 cells were seeded in 96-well plates at a density of 10^4^ cells/well. The next day, the cells were treated with 12.5 μM iCRT14. The cells were collected at various times, and cell viability was determined using the CellTiter-Glo® Luminescent Cell Viability Assay (Promega, Madison, WI, USA). This assay measures the relative number of viable cells based on the metabolic activity of the cells by quantifying cellular ATP content by a luminescent signal.

### RNA isolation and quantitative real-time PCR

Total RNA was extracted using TRI Reagent (Molecular Research Center, Cincinnati, OH, USA) and treated with DNase (Ambion, Grand Island, NY, USA). For host mRNA, 1 μg of RNA was reverse-transcribed into cDNA using oligo(dT) and random primers. Glyceraldehyde-3-phosphate dehydrogenase (GAPDH) was used as a housekeeping gene control. The various types of viral RNAs were quantified as described [2]. One microgram of RNA was reverse-transcribed using strand- and sense-specific primers for vRNA (5’-AGCGAAAGCAGG-3’ and 5’-AGCAAAAGCAGG-3’), cRNA (5’-AGTAGAAACAAGG-3’), and mRNA (oligo(dT)). GAPDH-specific primer (5’-GAAGATGGTGATGGGATTTC-3’) was added to all reverse transcription reactions. The primers used in this study are listed in Table 2. cDNA was diluted to 1:200 in nuclease-free water. Real-time PCR was carried out in a 20-μl reaction mixture containing specific primers and SYBR Green DNA dye (AnaSpec, Fremont, CA, USA). PCR was performed on a 7900HT Fast Real-Time PCR System (Applied Biosystems, Foster City, CA, USA) at cycling conditions of 95°C for 2 min and 40 cycles of 95°C for 15 s and 60°C for 60 s. Viral RNA levels, expressed as threshold cycle (*C*_*T*_) values, were normalized to GAPDH levels using the ΔCT method.

**Table 2:** Real-time PCR primers.

Gene Name		Sequence
NS	Forward	5’-CAAAAGCAGGGTGACAAAGACA-3’
	Reverse	5’-CTTGGTCTGCAACTCGTTTGC-3’
PB1	Forward	5’-GTCGAAAGGCTAAAGCATGGA-3’
	Reverse	5’-TGGCACTGAGATCTGCATGAC-3’
PB2	Forward	5’-CCGATGCCATAGAGGTGACA-3’
	Reverse	5’-GGAGACCAGCAGTCCAGCTTT-3’
PA	Forward	5’-GAAGTGCCATAGGCCAGGTTT-3’
	Reverse	5’-CAACGCCTCATCTCCATTCC-3’
NA	Forward	5’-TGTTGATGGAGCAAACGGAGTA-3’
	Reverse	5’-CTCAAACCCATGTCTGGAACTG-3’
NP	Forward	5’-TGTGTATGGACCTGCCGTAGC-3’
	Reverse	5’-CCATCCACACCAGTTGACTCTTG-3’
MP	Forward	5’-CTTCTAACCGAGGTCGAAACGTA-3’
	Reverse	5’-GGTGACAGGATTGGTCTTGTCTTTA-3’
HA	Forward	5’-GGCCCAACCACAACACAAAC-3’
	Reverse	5’-AGCCCTCCTTCTCCGTCAGC-3’
GAPDH	Forward	5’-GAAGGTGAAGGTCGGAGTC-3’
	Reverse	5’-GAAGATGGTGATGGGATTTC-3’
IFNβ1	Forward	5’-ATGACCAACAAGTGTCTCCTCC-3’
	Reverse	5’-GGAATCCAAGCAAGTTGTAGCTC-3'
IFNα1	Forward	5’-GCCTCGCCCTTTGCTTTACT-3’
	Reverse	5’-CTGTGGGTCTCAGGGAGATCA-3’
18S rRNA	Forward	5’-CGTTGATTAAGTCCCTGCCCTT-3’
	Reverse	5’-TCAAGTTCGACCGTCTTCTCAG-3’

### Animal studies

The animal procedures were approved by the Institutional Animal Care and Use Committee (IACUC) at Oklahoma State University. Eight-week-old female C57BL/6J mice (Jackson Laboratory, Bar Harbor, ME) were used in this study. Animal housing and environmental conditions met all applicable standards. The animals were anesthetized with intraperitoneal injection of ketamine (100 mg/kg) and xylazine (10 mg/kg). The mice were infected intranasally with 1,000 plaque forming unit (pfu) (for a lethal challenge) or with 100 pfu (for a sublethal challenge) of influenza A virus (A/PR/8/34) in 50 μl volume. The mice received intraperitoneal injections of iCRT14 (50 mg/kg body weight) or of DMSO (4.2%) [46] as a vehicle control in a volume of 800 μl one day prior to virus infection and then daily from day 2 to day 5, as indicated. The mice were monitored for clinical signs such as arching back, huddling and ruffled fur. Body weight loss was monitored daily; animal health and behavior were frequently observed by animal caretaker; mice that lost more than 30% of their original body weights were euthanized by injection of ketamine (100 mg/kg) and xylazine (10 mg/kg) followed by cervical dislocation. For sublethal studies, clinical signs were scored as previously described [47]: normal = 0, ruffled fur = 1, inactive = 3, hunched back and moribund = 4. Mice were euthanized at day 2 or day 5 after infection. The left lungs of the animals were fixed in 10% neutral buffered formalin (Thermo Scientific, West Palm Beach, FL, USA); the right lungs were snap-frozen in liquid nitrogen and used for RNA, protein and virus titer determination. For determination of virus titer in lung tissue, the right cranial lung lobes of infected mice were homogenized in 10% (w/v) phosphate-buffered saline (PBS) and used in TCID_50_ assays.

In a separate experiment, the right lungs of the animals were used for collection of broncheoalveolar lavage (BAL), and the left lungs were used to determine the wet-to-dry ratio. The right lungs were lavaged twice with 500 μl ice-cold PBS; approximately 80% of the applied volume was recovered. The BAL cells were centrifuged, resuspended and cytospin to slides. The slides were stained with Dip Quick stain (Jorvet, Loveland, CO, USA). Differential cell counts were performed using ≥ 400 cells per sample. The BAL fluid was frozen until use.

### Lactate dehydrogenase (LDH) assay and total protein measurement

Lavage from the right lung lobe of each mouse in each group was used for LDH assay and total protein measurement. Fifty-microliter aliquots were used to measure the activity of LDH by monitoring the reduction of nicotinamide adenine dinucleotide at 340 nm in the presence of lactate using a Thermo Scientific (Rockford, IL, USA) LDH assay kit. Total protein in the BAL fluid was estimated by a modified Bradford assay (Bio-Rad, Hercules, CA, USA) according to the manufacturer's instructions; the remainder of the fluid was frozen at −80°C until processed.

### Histopathologic analyses

Animals were euthanized by exsanguination through the abdominal aorta under xylazine and ketamine anesthesia following an approved IACUC protocol. Formaline-perfused lungs were embedded in paraffin wax. Sections 4 μm in thickness were cut and stained with hematoxylin and eosin (H&E). Histopathologic lesions were scored by a board-certified pathologist as described [48]: 1 = minimal damage to alveolar structures; 2 = mild; 3 = moderate; and 4 = marked/severe damage to lung tissue.

### Topflash luciferase assay

HEK293 cells were cotransfected with a TOPflash reporter plasmid (20 ng), which contains three copies of LEF binding sites upstream of the thiamine kinase (TK) minimal promoter and firefly luciferase gene (EMD Millipore, Billerica, MA, USA) and a pRL-TK vector (1 ng) for normalization. Twenty four h post transfection, the cells were infected with A/WSN/33 or A/PR/8/34 at an MOI of 0.1 or left un-infected. Firefly and *Renilla* luciferase activities were measured using the Dual-luciferase Reporter Assay Kit (Promega, Madison, WI, USA).

### Influenza virus reporter assay

To detect influenza A virus replication, we constructed an influenza A luciferase reporter vector encoding a firefly luciferase under the control of NP 5’ and 3’ UTRs of influenza A/WSN/33 according to a previous report [49]. The firefly luciferase gene was inserted into the RNA polymerase I expression vector, pHH21 (a kind gift from Dr. Yoshi Kawaoka, University of Wisconsin) through BsmB I sites (NP-UTR-Luc). A549 cells were cotrasnfected with the virus sensor, NP-UTR-Luc (20 ng) and a pRL-TK vector (20 ng). Twenty four h post transfection, the cells were infected with A/PR/8/34, A/WSN/33, pdm/Ok/09, A/Wisconsin/67/05 at an MOI of 0.1. Forty eight h post infection, luciferase activities were measured using the Dual-luciferase Reporter Assay Kit (Promega, Madison, WI, USA).

### Data analysis

The data from at least three independent experiments were analyzed. The results were statistically analyzed using Student's t-test, one-way or two-way ANOVA followed by post hoc Tukey or sidak test using Graph Pad Prism version 6.0. *P* values of <0.05 were considered significant.

## Supporting information

S1 File Plos-one-human-endpoints-checklist.doc. (DOCX)
